# Key considerations for conducting Chinese medicine clinical trials in hospitals

**DOI:** 10.1186/1749-8546-8-3

**Published:** 2013-02-16

**Authors:** Johannah L Shergis, Shefton Parker, Meaghan E Coyle, Anthony L Zhang, Charlie C Xue

**Affiliations:** 1 Traditional and Complementary Medicine Research Program, School of Health Sciences and Health Innovations Research Institute (HIRi), RMIT University, PO Box 71, Bundoora, Victoria 3083, Australia

## Abstract

Conducting clinical trials of Chinese medicines (CM) in hospitals presents challenges for researchers. The success of hospital-based CM clinical trials may be influenced by the protocol design, including the maintenance of CM theory in compliance with scientific rigour and hospital guidelines and justified treatment approaches with results that can translate into clinical practice. Other influences include personnel and resources such as a dedicated team open to CM with an established research culture and the ability to maximise participant recruitment. This article identifies the key challenges and limitations of conducting CM clinical trials in Australian hospitals.

## Commentary

Chinese medicine (CM) treatments, including acupuncture, herbal medicine, remedial manual therapy, and exercise therapy (*Tai Chi*) are growing in popularity [1-3]. At the same time the healthcare community have begun to acknowledge the preferences of patients to receive treatment with CM in the United Kingdom, United States and Australia [4-7]. In Australia, CM practitioners must be registered nationally by the Chinese Medicine Board of Australia through the Australian Health Practitioners Regulation Agency in order to practice acupuncture and prescribe Chinese herbal medicine [8].

The practice and paradigmatic philosophies of CM differ from those of Western medicine (WM). Traditional CM diagnostic methods consider factors such as the environment, seasons, emotions, diet, and lifestyle. In addition WM diagnostic approaches are also used by CM practitioners to provide a comprehensive diagnosis and individualised treatment plan. In contrast, WM focuses on pathological processes and uses diagnostic instruments or tests or both to classify illness, and places less of an emphasis on a person’s interaction with the environment. These differences may create gaps in communication between the disciplines; however, recently cooperation has grown [9].

Evidence-based medicine (EBM) including randomized controlled trials (RCT) is likely to bridge the gap further between CM and WM. Increasing numbers of RCTs are being undertaken to determine the efficacy and safety of CM interventions [10]. CM researchers have been striving to achieve high standards of scientific rigor through RCTs. However, this has presented challenges in harmonizing CM theory and the RCT design required to enable effective translation [11].

Given the relatively short history of RCTs in CM outside China, a substantial proportion of CM studies were usually conducted in universities or community clinics. In Australia, hospitals are increasingly involved in clinical studies since the introduction of national strategies and processes to encourage greater engagement and participation of hospitals in research [12]. A search of the Australian and New Zealand Clinical Trials Registry (ANZCTR) on 23 August 2012 identified 144 alternative and complementary medicine RCTs with ethical approval being conducted in Australia. Of these, 71 were CM studies, and 16 were identified as being conducted in hospitals. The availability of participants (*e.g.*, acute, sub-acute and outpatients), easy access to hospital resources (*e.g.*, functional magnetic resonance imaging, fMRI) and medical expertise are major factors which make hospitals suitable environments for undertaking CM clinical trials.

As a typical example, this paper will highlight some of the challenges of undertaking RCTs in CM interventions, based on our multi-centre hospital studies in Melbourne, Australia (Table 1). The Multiple Emergency Department Acupuncture Trial (MEDACT) evaluated the analgesic effect of acupuncture for patients presenting to the emergency department (ED) with low back pain, ankle sprain or migraine [13]. This study was pragmatic in design and allocated participants randomly to one of three groups: acupuncture alone; acupuncture plus pharmacotherapy; and pharmacotherapy alone. Recruitment occurred in four EDs and the primary outcome was pain relief after one hour. The Ginseng Extract and Respiratory Symptoms (GEARS) is ongoing, and is evaluating the efficacy of a herbal medicine in participants with chronic obstructive pulmonary disease (COPD) [14]. This study is a two-arm parallel RCT and participants are recruited through four metropolitan hospitals. This paper will discuss the issues we encountered, including protocol design, personnel and resources, and participant recruitment.

**Table 1:** Characteristics of the MEDACT and GEARS clinical trials

	**MEDACT**	**GEARS**
**Condition treated**	Acute pain; ankle sprain, non-specific low back pain or migraine	COPD
**Design**	RCT	RCT
**Funding source**	Government funding body (NHMRC)	Government funding body (NHMRC)
**Source of patients**	Hospital ED	Community and outpatients
**Location of patient visits**	Hospital ED	Hospital outpatients
**Number of sites**	4	4
**Number of participants**	505	168
**Number of groups**	3	2
**Intervention**	Acupuncture or acupuncture plus standard care (pharmacotherapy)	*Panax ginseng *(herb)
**Control**	Standard care (pharmacotherapy)	Placebo
**Treatment duration**	1 treatment	6 months
**Follow-up**	48 hours	6 months
**Outcomes**	1. Pain and disability scales	1. Quality of life
	2. Quality of life	2. Lung function
	3. Acceptably of treatment and willingness to repeat	3. Use of relief medication
	4. Adverse events	4. Exacerbations
	5. Health resource utilization	5. Adverse events
**Recruitment status**	Completed: target reached 2011	Ongoing: completion target 2013

COPD: Chronic obstructive pulmonary disease, ED: Emergency department, GEARS: Ginseng extract and respiratory symptoms clinical trial, MEDACT: Multiple emergency department acupuncture control trial, NHMRC: National Health and Medical Research Council, RCT: Randomized controlled trial.

### Protocol design

RCTs should meet standards of rigorous scientific design. RCTs need to incorporate design elements to maintain internal and external validity for generalisability. In Australian hospitals, CM treatments are not prescribed routinely; thus, researchers need to ensure that the trial is implemented in accordance with hospital policies and guidelines without compromising the quality of the research. Furthermore, in order to be clinically relevant, the results of the trial should be translatable to a clinical practice setting.

A major consideration in designing a hospital-based CM RCT is the study design. Feasible protocols are required to implement and researchers need to consider the appropriateness and justification for complex individualised interventions and non-standard treatments, particularly when using herbal medicines [15].

Another main consideration for CM studies is the justification of intervention. This is determined predominantly through historical use and is usually supported by records with pharmacological and toxicological reports and detailed information of the herbal product’s constituents. Acupuncture studies may encounter similar questions regarding evidence base and mechanism of action, to justify its use for a particular health condition.

Traditional CM diagnosis and treatment are based on interview and observation of the patient, and do not require instrumental tests, as in WM. When a WM diagnosis is required to assess eligibility for inclusion in a pragmatic trial, and the CM diagnosis is used only to guide selection of an appropriate treatment for each individual, the trial may be better suited to the hospital setting. For example, the MEDACT study required the WM diagnosis of an ankle sprain, migraine or low back pain [13]. Although a CM diagnosis was not an inclusion criterion, the acupuncturists were able to select appropriate acupuncture points based on CM theory.

In contrast, the GEARS study included participants according to the WM diagnosis of COPD in addition to the CM diagnosis of *lung qi* deficiency or *spleen qi* deficiency, or both. The intervention in this study was a herb used to treat *lung qi* deficiency or *spleen qi* deficiency, or both [14]. The WM and CM diagnoses enabled the translation of the outcomes into clinical practices.

The RCTs that require specialist testing and diagnostic equipment to confirm a diagnosis may be better suited to the hospital setting. Through negotiation with relevant hospital departments, access to testing and equipment may facilitate trial recruitment and reduce costs. Diagnostic X-ray was often required in the MEDACT study to exclude injuries caused by fracture. In the GEARS study, lung function testing was undertaken using spirometry, hospital pathology laboratories performed full blood examinations, including tests of liver and kidney function and trial medications were dispensed from the hospital pharmacy.

### Personnel and resources

Conducting a high standard clinical trial requires a dedicated and experienced research team. Skilled investigators and trial coordinators are frequently employed by hospital research departments and are essential in a successful study [16]. The culture of an organisation and their openness to CM theory will aid in the conduct of the trial and contribute to recruitment success [17]. A hospital setting would be counterproductive if researchers in hospitals are not enthusiastic in CM research projects.

In the MEDACT study, ED doctors confirmed eligibility of participants then qualified acupuncturists delivered the acupuncture treatments. In the GEARS study, a qualified CM practitioner was employed during the screening process to ensure the participants met the pre-specified CM diagnostic criteria. All other activities, including randomisation, were completed by a clinical trial coordinator employed by the hospitals.

Study sites should be accessible to trial participants and responsive to queries if problems arise. Hospitals often have an established research culture and can offer a setting that improves the running of trials by providing the facilities required to ensure ‘Good Clinical Practice’ (GCP). Other advantages include the availability of facilities (*e.g.*, radiology and pathology equipment) and trained specialized staff. Hospital based pharmacy departments are often a convenient location for storing and dispensing study medications, which assist in maintaining the integrity of blinding. Availability of these facilities was crucial to the conduct of both MEDACT and GEARS.

Hospitals have the potential to be more expensive than universities or clinic settings for conducting RCTs when taking into account the costs of site-set up, ongoing fees, personnel, per-recruitment charges and the legal costs of clinical trial agreements. This was found to be the case for the GEARS study. The study incurred approximately $5,500 (Australian dollars) of additional hospital fees per site. This included ethics, site-set up, administration fees, ongoing fees, and additional per-recruitment charges of up to $250. The MEDACT study also incurred similar costs.

### Participant recruitment

Recruitment and retention of participants in clinical trials can be challenging due to trial-specific demands, the availability of patients, the preferences of patients and informed consent [18]. Individuals may participate in CM studies just as willingly as WM studies [19]; however, there is a dearth of research evaluating how the trial site might influence study participation.

The attitudes of potential participants towards CM clinical trials also influence recruitment [17,20]. Positive attitudes in recruitment for CM clinical trials include: (1) CM is considered safer and less invasive than WM; (2) CM treatments may help to reduce dependence on conventional medications; and (3) the willingness of participants to try new interventions at little cost or harm to themselves. By contrast, barriers to recruitment include: (1) doubt about the effectiveness of treatments; (2) lack of knowledge of how CM may work; (3) participants are not willing to change their current health care options and reluctant for randomisation when CM treatments are readily available in the community.

In the MEDACT study, patients were approached for screening soon after triage by the ED nurse. An early approach appeared to improve the chances of participation. The steady flow of patients through the ED combined with the motivation of these patients to obtain pain relief enabled satisfactory recruitment within a relatively short period. Little advertisement or incentive saved costs and enabled staff to concentrate on the ED during peak times in order to maximise recruitment.

A different recruitment strategy was used for the GEARS study as patients with stage II COPD were more likely to be outpatients. The recruitment strategy included media promotion, advertising in clinic rooms, and physician referrals. As this trial has not been completed, we have not yet evaluated the recruitment strategy.

## Conclusion

The appropriateness of conducting a hospital-based CM clinical trial depends on the requirements for the clinical setting. We identified several key elements to consider before conducting a CM RCT in a hospital, including protocol design; personnel and resources; and participant recruitment.

## Abbreviations

ANZCTR: Australian and New Zealand clinical trials registry; CM: Chinese medicine; COPD: Chronic obstructive pulmonary disease; EBM: Evidence-based medicine; ED: Emergency department; GCP: Good clinical practice; GEARS: Ginseng extract and respiratory symptoms; MEDACT: Multiple emergency department acupuncture trial; RCT: Randomized controlled trial; WM: Western medicine.

## Competing interests

The authors declare that they have no competing interests.

## Authors’ contributions

JS, SP and CX determined the manuscript scope and information. JS, SP, MC, AZ and CX wrote the manuscript. All authors read and approved the final version of the manuscript.
